# Supplementary material for: The epidemiology of SARS-CoV-2, Influenza, and Respiratory Syncytial Virus circulation among adult patients with acute respiratory illness in Kampala Metropolitan Area in Uganda
Source: PLoS One. 2026 May 21;21(5):e0344869. doi: 10.1371/journal.pone.0344869 (PMC13193385; doi:10.1371/journal.pone.0344869)
Supplement: Attachment — Submitted filename: Response to Reviewers 11thJan2026.pdf [file pone.0344869.s002.pdf]

Makerere University
Makerere University Lung Institute
Makerere University College of Health Sciences, New Mulago Hospital Complex
Upper Mulago Hill Road. P.O. Box 7749, Kampala Uganda
Email : mli@chs.mak.ac.ug Tel : +256 (0) 414 699 134 Web : http://mli.mak.ac.ug
mli

11th Jan 2026

Response to Reviewers

Manuscript ID: PONE-D-25-42419

Title: The epidemiology of SARS-CoV-2, Influenza, and Respiratory Syncytial Virus circulation among adult patients with acute respiratory infection in Kampala Metropolitan Area in Uganda

Dear Academic Editor and Reviewers,

We sincerely thank the Academic Editor and the reviewers for their thoughtful evaluation of our manuscript and for the constructive comments provided. We have carefully revised the manuscript to address all concerns raised. Below, we provide a detailed, point-by-point response to each comment. All changes have been incorporated into the revised manuscript, with sections clarified, reorganized, or corrected as appropriate.

Responses to the Academic Editor and Journal Requirements

1. PLOS ONE formatting and file requirements

Response: The manuscript has been revised to fully comply with PLOS ONE formatting guidelines. Tables are now embedded in the main manuscript, supplementary materials are provided as supporting information files, and file naming conventions have been corrected.

2. Role of the funder: Comment: Please state the role of the funders.

Response: We have added the following explicit statement to both the manuscript and the cover letter: “The funders had no role in study design, data collection and analysis, decision to publish, or preparation of the manuscript.”

3–4. Data availability and open data policy; Comment: Authors must clarify the data sharing plan and compliance with PLOS open data requirements.

Response: The datasets generated and analyzed during this study will be made available upon acceptance of the manuscript. De-identified individual-level data will be deposited in a publicly accessible repository, subject to compliance with the ethical approvals granted by the Mulago Research and Ethics Committee and the Uganda National Council for Science and Technology.

5. Ethics statement location

Response: The ethics statement has been retained exclusively under declaration section. All duplicate references elsewhere in the manuscript have been removed.

6. Tables

Response: Tables well aligned in the manuscript. Table 1 is under subsection, **Participant characteristics,** which starts with *“Out of the total1,265 participants………”* Table 2 is under subsection **Clinical and occupational correlates**. With starts with statement “*Out of 1,265 participants screened, In the 510 participants with complete clinical and occupational data……………..”*. Figure 1 is under section **Seasonal trends** which starts with statement **“***Epidemiological time-series analysis of the 13-month surveillance period…..”* <u>Line 222, Line 232 and Line 251 of clean copy respectively</u>

7. Citation recommendations

Response: All suggested references were reviewed, and relevant citations were included where appropriate.

Reviewer #1

Comments: Comment (Lines 115–116): Influenza pandemic statement

Response: We revised the wording to avoid implying that influenza viruses no longer pose pandemic risks. The revised text acknowledges the continued pandemic potential of influenza viruses.

Comment (Lines 150–152): Exclusion of hospitalized patients and pregnant/lactating women

Response: We clarified that these exclusions were due to the parent clinical trial design. This justification is now clearly stated in the Methods section, and the resulting limitations including potential selection bias are discussed explicitly in the Study Limitations subsection.

Comment (Lines 189–191): Univariable vs multivariable analysis

Response: This inconsistency has been corrected. The Methods section now clearly states that both univariable and multivariable logistic regression analyses were conducted.

Comment (Lines 246–248): Healthcare worker bias

Response: We expanded the Discussion to acknowledge that increased healthcare access among healthcare workers may partly explain the higher observed SARS-CoV-2 prevalence, in addition to occupational exposure.

Comment (Line 259): Vaccination type

Response: We clarified that vaccination status refers specifically to SARS-CoV-2 vaccination, as no influenza or RSV vaccination was administered during the study period.

Comment (Line 265): Sentence placement

Response: The sentence referring to exposure bias rather than vaccine failure has been moved to the Discussion section.

Comment (Line 274): Influenza terminology

Response: Terminology has been standardized throughout the manuscript to consistently use "Influenza A" and "Influenza B."

Comment (Line 280): Apparent contradiction regarding sore throat

Response: We revised the text to clarify that while influenza A and B infections were more frequently detected among participants reporting sore throat, sore throat was highly prevalent overall and therefore not a distinguishing clinical feature of influenza infection.

Comment (Line 320): SARS-CoV-2 as a bystander

Response: We incorporated this interpretation into the Discussion, acknowledging that SARS-CoV-2 may have been detected as a coincidental finding in vaccinated symptomatic individuals with other unidentified infections.

Comment (Lines 347–350): Missing data bias

Response: We added a statement discussing potential differential bias due to missing data and noted that missingness was not systematically associated with age or sex.

Comment (Line 357+): Implications for vaccination strategies

Response: We added a concise paragraph discussing implications for vaccination strategies in Uganda, emphasizing risk-based prioritization of older adults and healthcare workers and timing vaccination ahead of observed peaks in virus circulation.

Comment (Line 372): Typographical error

Response: The spelling error ("prepweredness") has been corrected.

Comment (Table 1): Coinfections

Response: We have added a description of observed coinfections in the Results section and referenced these findings in the text.

Comment (Table 2): Missing data and totals

Response: Table 2 now includes totals out of 510 participants, and missing data are clearly indicated and explained in the table and Results section.

Reviewer #2 Comments

Comment: Study design classification

Response: We revised the Methods section to clearly justify classification as an observational cross sectional design conducted over a 13-month period, from March 2023 to April 2024.

Comment (Lines 228–232): Table justification in Methods

Response: All explanations justifying Tables 1 and 2 were removed from the Methods section to avoid redundancy with the Results section.

Comment: Results structure and multivariable analysis

Response: We merged and streamlined the indicated Results paragraphs, ensuring that this section focuses strictly on findings. Interpretative statements were moved to the Discussion.

Comment: Seasonal trends vs clinical correlates

Response: The Results section was reorganized to clearly separate seasonal trends from clinical and occupational correlates, eliminating repetition.

Comment: Conclusion length and limitations

Response: The Conclusion has been shortened substantially, and study limitations are now presented in a dedicated paragraph, as recommended.

Closing Statement

We believe that the revisions have significantly strengthened the manuscript and fully addressed the concerns raised by the Academic Editor and reviewers. We are grateful for the insightful feedback and hope that the revised manuscript is now suitable for publication in PLOS ONE.

Sincerely,

Sincerely,

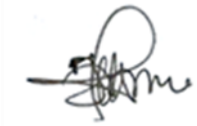

James Arinaitwe on behalf of all authors
Email: arinaitwejim@gmail.com
Tel. +256785142165
Author

for **Corresponding Author:**
Professor Bruce J. Kirenga
Director, Makerere University Lung Institute
Email: brucekirenga@yahoo.co.uk
