# Supplementary material for: The epidemiology of SARS-CoV-2, Influenza, and Respiratory Syncytial Virus circulation among adult patients with acute respiratory illness in Kampala Metropolitan Area in Uganda
Source: PLoS One. 2026 May 21;21(5):e0344869. doi: 10.1371/journal.pone.0344869 (PMC13193385; doi:10.1371/journal.pone.0344869)
Supplement: Attachment — Submitted filename: Response to Editor 17thFeb2026.pdf [file pone.0344869.s004.pdf]

Makerere University

Makerere University Lung Institute

Makerere University College of Health Sciences, New Mulago Hospital Complex
Upper Mulago Hill Road. P.O. Box 7749, Kampala Uganda
Email : **mli@chs.mak.ac.ug** Tel : **+256 (0) 414 699 134** Web : **http://mli.mak.ac.ug**

mli

17th Feb 2026

Editor-in-Chief
PLOS ONE

Dear Editor,

RE: RESPONSE TO EDITOR: MANUSCRIPT ID: PONE-D-25-42419

We sincerely thank you and all reviewers for the careful evaluation of our revised manuscript entitled: **"The epidemiology of SARS-CoV-2, Influenza, and Respiratory Syncytial Virus circulation among adult patients with acute respiratory infection in Kampala Metropolitan Area in Uganda."**

We sincerely appreciate prompt feedback. Below, we provide our response to the guidance given in the latest email.

Editor #1 Comment:

"Please include your tables as part of your main manuscript and remove the individual files. Please note that supplementary tables (should remain/ be uploaded) as separate "Supporting Information" files."

Response:

Table 1 has been inserted into the main manuscript from Line 232 to Line 234

Figure 1 has been inserted into the main Manuscript from lines 242 to 244

Table 2 has been inserted into the main manuscript from Line 269 to Line 271

All previous submissions has been removed as per guidance

Sincerely,

James Arinaitwe on behalf of all authors
Email: arinaitwejim@gmail.com
Tel. +256785142165
Author

for : **Corresponding Author:**
Professor Bruce J. Kirenga
Director, Makerere University Lung Institute
Email: brucekirenga@yahoo.co.uk
