# Supplementary material for: The epidemiology of SARS-CoV-2, Influenza, and Respiratory Syncytial Virus circulation among adult patients with acute respiratory illness in Kampala Metropolitan Area in Uganda
Source: PLoS One. 2026 May 21;21(5):e0344869. doi: 10.1371/journal.pone.0344869 (PMC13193385; doi:10.1371/journal.pone.0344869)
Supplement: Attachment — Submitted filename: Review.docx [file pone.0344869.s001.docx]

Review (PLOS One)

The authors said that “acute respiratory infections (ARI) caused by viruses such as SARS-CoV-2, influenza, and respiratory syncytial virus (RSV) posed significant public health challenges, particularly in low-income countries. Understanding the co-circulation dynamics of these pathogens is crucial for effective public health monitoring, surveillance, and control interventions”. The authors have raised an important issue with the aim of characterizing the epidemiologic dynamics of SARS-CoV-2, influenza, and RSV co-circulation among participants with ARI in a post-COVID-19 pandemic setting in Uganda's central urban and peri-urban districts business districts.

However, there are some concerns:

**Methodology**

Based on the methodology used, I do not understand why the authors qualified this study as a retrospective cohort study. This should clearly be explained.

Line 228-232: The clarifications to justify Table 2 and Table 1 should not be done in this section since they have been repeated in the result section.

**Results**

Line 244 -260 : these two paragraphs should be combined since the Multivariate analysis is only **a** technical tool empowering the observed results. Additionally in this section all sentences related to explain some results are note expected (e.g. line 266).

Lines 274-285: this paragraph does not fit with the “Seasonal trends” paragraph. May be the authors should reorganize all these information’s in the “**Clinical and occupational correlates”** by avoiding repetition of the same information’s (e.g. Lines 246,247, 248-274, 275).

**Conclusion**

The conclusion should be shortly formulated and possible limitations of the study should be stated in a special paragraph.
