# Supplementary material for: The epidemiology of SARS-CoV-2, Influenza, and Respiratory Syncytial Virus circulation among adult patients with acute respiratory illness in Kampala Metropolitan Area in Uganda
Source: PLoS One. 2026 May 21;21(5):e0344869. doi: 10.1371/journal.pone.0344869 (PMC13193385; doi:10.1371/journal.pone.0344869)
Supplement: Attachment — Submitted filename: Review 1.docx [file pone.0344869.s003.docx]

Review 1 (PLOS One)

All the comments/edits raised have been attended. I have no further comments
